# Body Image in BRCA-Positive Young Women Following Bilateral Risk-Reducing Mastectomy: A Review of the Literature

**DOI:** 10.3389/fpsyg.2021.778484

**Published:** 2021-12-16

**Authors:** Christa Torrisi

**Affiliations:** Sinclair School of Nursing, University of Missouri-Columbia, Columbia, MO, United States

**Keywords:** body image, BRCA, risk-reducing mastectomy, prophylactic mastectomy, young adult, risk-reducing surgery

## Abstract

**Background/Significance:** The presence of a breast cancer (BRCA) gene mutation increases a woman’s lifetime risk of developing breast cancer. Bilateral risk-reducing mastectomy is a proactive treatment option which lowers that risk. However, breast removal can create a change in physical appearance. It is unclear if BRCA-positive women undergoing this surgery in young adulthood, a life stage where intimate relationships, families, and careers are being established, have the same experience with body image as women in later stages of life.

**Purpose:** The aim of this literature review is to assess how bilateral risk-reducing mastectomy impacts body image in young BRCA-positive women less than 40 years of age, with no history of breast cancer.

**Methods:** Database searches were performed, yielding 402 results. Studies were considered if participants had an increased lifetime breast cancer risk/BRCA-positive diagnosis and history of bilateral risk-reducing mastectomy, body image was evaluated, and mean age was less than 40 years. A total of three qualitative studies and three quantitative studies were identified as relevant for this review.

**Results:** A dearth of information exists on body image in young women following bilateral risk-reducing mastectomy. It was found in this review that some women experienced a decline in body image following surgery, while in others body image was maintained or improved.

**Conclusion:** Understanding factors that impact body image following this risk-reducing surgery will allow clinicians to support this unique population. Open and informative discussion should be encouraged with young women considering, or who have undergone, bilateral risk-reducing mastectomy.

## Introduction

The presence of a breast cancer (BRCA) gene mutation is predicted to increase a woman’s lifetime risk of developing breast cancer to 72%, compared to the 12% lifetime risk of a woman without this gene mutation (Kuchenbaecker et al., 2017; McCarthy et al., 2017). Approximately, 1 in 400 people in the United States have a BRCA 1 or 2 mutation (National Cancer Institute, 2020). Women less than 40 years of age have accounted for a 15% increase in BRCA testing between 2003 and 2016 (Guo et al., 2020), emphasizing the importance of knowing and managing risk to this population.

Bilateral risk-reducing mastectomy (BRRM), a surgical procedure in which healthy breasts are removed to prevent breast carcinoma, is the most effective proactive treatment option available for BRCA-positive women. Lifetime breast cancer risk is reduced by 90% following BRRM (Rebbeck et al., 2004). This procedure continues to increase in prevalence, with insurance claims database surveys between 2003 and 2016 displaying that BRCA-positive women undergoing BRRM had increased between 1.2 and 1.6% per month (Liede et al., 2018).

Younger women who undergo BRRM note having a close family member diagnosed with breast cancer and the reduction of breast cancer-related worry as common reasons for seeking this surgery (Hoskins and Greene, 2012; Borreani et al., 2014; Hunt et al., 2017; Long et al., 2017; Henry et al., 2019), despite its potential to cause a significant change in appearance. Body image, defined as a person’s attitudes, thoughts, beliefs, and behaviors regarding their physical appearance (Cash, 2004), has been negatively impacted by mastectomy in young breast cancer survivors (Iddrisu et al., 2020; Rosenberg et al., 2020). Body image has been both positively and negatively affected following BRRM in studies comprised of women across the lifespan (Metcalfe et al., 2015; Razdan et al., 2016; Bai et al., 2019). Younger women are undergoing this surgery during a stage in life where commitments to careers, families, and/or monogamous relationships are being established (Scheck, 2005), and may be affected differently than women 40 years of age or older. The aim of this review of the literature is to examine how BRRM effects body image in BRCA-positive women less than 40 years of age.

## Materials and Methods

### Search Strategy

Searches were performed 06/16/2021, and 08/22/2021 utilizing a combination of the key terms of “risk-reducing mastectomy,” “BRCA,” “body image,” and “young.” CINAHL, PubMed, and Scopus databases were utilized in the review of literature. No limits were placed on geographic location or length of time for follow up. Studies were limited to those published in peer reviewed journals from 2011 and onward, and in the English language. This timeframe for article selection was chosen as the use of prophylactic breast surgery to reduce lifetime breast cancer risk in young women is a relatively new concept.

### Eligibility Criteria

Studies selected for this review included those in which participants had previously undergone BRRM, were BRCA-positive or had an increased familial risk of breast cancer, and those in which body image was addressed within the study. Articles were included if body image broadly met the definition provided in this review. Due to the paucity of studies comprised solely of participants less than 40 years of age, studies were considered for inclusion if the mean age of study participants was less than 40 years. Studies were also included if data on women who had undergone BRRM were reported separately from women undergoing other risk-reducing surgery or who were breast cancer survivors. Two qualitative articles used data collected from semi-structured interviews in the same sample of participants. From this study, both articles were included as each pursued different research questions and aims (Glassey et al., 2018b). Ineligible studies were those in which mean age of participants was greater than 40 years and were comprised of, or did not separately report data from, women with a breast cancer diagnosis.

### Study Selection

**FIGURE 1:**
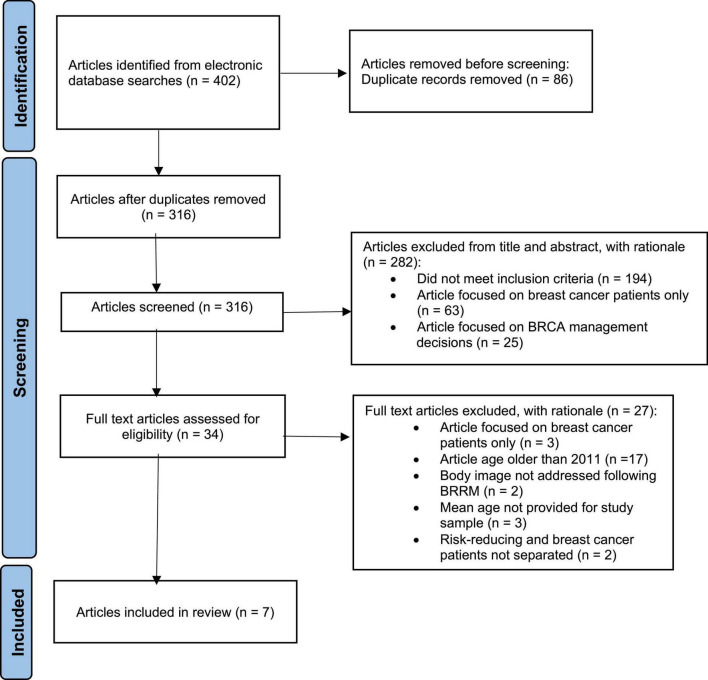
PRISMA flow diagram for article inclusion.

Searches yielded 402 results, with 316 articles remaining after duplicates were removed. Titles and abstracts of these results were then screened for relevance, with 34 articles identified for full text review. The articles were then read in their entirety and were excluded for the following reasons: three contained participants that were only breast cancer survivors; 17 had a mean age greater than 40 years; three did not provide a mean age for study participants; two did not address body image; and two did not differentiate participants with and without breast cancer. Seven articles were identified as meeting inclusion criteria for this literature review (see Figure 1).

## Results

### General Characteristics of Included Studies

Table 1 displays study characteristics for the seven articles included in this review. While all studies had a mean age of less than 40 years, only two studies contained participants who were all less than 40 years of age at the time of BRRM (Glassey et al., 2018b; Salibian et al., 2020). Studies were located across Europe (*n* = 3), Australia and New Zealand (*n* = 2), and the United States (*n* = 1). In all studies participants underwent some form of reconstruction following mastectomy, though details on reporting reconstruction type(s) varied widely across studies. Three quantitative articles were included in this review. All are observational, with participants completing surveys. Of the three qualitative studies included in this review, all conducted semi-structured interviews to obtain data. In analyzing this data, two articles, which used the same sample population, utilized interpretative phenomenological analysis theory (Glassey et al., 2018b), one study used thematic qualitative analysis (Hallowell et al., 2012), and one used content analysis (Wasteson et al., 2011).

**TABLE 1:** Table of studies.

First author/Study type (Year)	Design/Method	Sample/Setting	Inclusion criteria	Body image instrument/Items	Analysis/Results	Findings addressing body image
Gandhi et al. (2021)/Quantitative (2021)	• Observational, multi-site (2) • Data collected between 2015 and 2016 • The BREAST-Q reconstruction module was completed after BRRM to measure satisfaction with breasts	• *N* = 241 women without breast cancer • x̄ age of women without breast cancer at BRRM: 39.6 years • Location: United Kingdom	• Increased familial breast cancer risk • BRRM	• BREAST-Q reconstruction module/99 items	• Satisfaction with breasts score: 62 • Psychosocial well-being score: 73	• The BREAST-Q displayed high breast satisfaction and psychosocial well-being scores (out of 100) following BRRM
Glassey et al., 2018b/Qualitative (2018 and 2018)	• Interpretive phenomenological analysis • Data collected between 2015 and 2016 • One semi-structured interview was conducted per participant to explore whether psychological consultation prior to BRRM was beneficial to women • One semi-structured interview was conducted per participant to explore the influences on satisfaction with reconstructed breasts	• *N* = 26 • x̄ age at time of study = 31 years (range 23–38 years) • Location: Australia and New Zealand	• BRCA-positive diagnosis or strong family history of breast cancer • Age under 35 at time of BRRM	Semi-structured interview guide	Interpretative phenomenological analysis/3 themes surrounding psychological consult: psychological well-being and adjustment, intimacy, and body image and 4 themes surrounding breast satisfaction: satisfaction with breasts before surgery, outcome expectations, type of mastectomy, and open communication	• Women who underwent psychological evaluation (38%) prior to BRRM upheld their confidence and self-esteem following surgery; body image was maintained • Women who were satisfied with breast appearance prior to BRRM were less satisfied after • Unrealistic surgical expectations caused a decline in body image • Loss of nipples caused a decline in body image
Gopie et al., 2013/Quantitative (2013)	• Observational, multi-site, prospective • Data collected between 2007 and 2010 • Women filled out a study-specific body image survey preoperatively, 6 months postoperatively, and after reconstruction to determine the psychological impact of breast reconstruction	• *N* = 73 • x̄ age at time of BRRM = 37.1 years (range at time of BRRM: 21–65 years) • Location: Netherlands	• BRCA-positive diagnosis or increased familiar breast cancer risk	• Study specific Body Image Scale (BIS)/31 items • Impact of Event Scale (IES)/15 items	Cohen’s *d* = −0.63 at 6 months Cohen’s *d* = −0.83 at 21 months	• A high preoperative cancer distress score led to a more negative body image at long term follow-up in 25% (*n* = 12) of the women • Discussion of outcomes is important for expectation management
Hallowell et al. (2012)/Qualitative (2012)	• Thematic Analysis • Data collected between 2006 and 2009 • One semi-structured interview was conducted per participant to explore the experiences of women 3 years after risk-reducing surgery, including BRRM	• *N* = 8 • x̄ age at time of BRRM: 34 years (range at time of BRRM: 28–41 years) • Location: Australia and New Zealand	• No previous breast cancer diagnosis • BRCA-positive diagnosis or increased familial risk of breast cancer	Semi-structured interview guide	Thematic analysis/2 themes: looking different, feeling different	• Positive and negative impressions were experienced by women following BRRM • BRRM offered cosmetic improvement to some participants who were unhappy with their appearance prior to surgery
Salibian et al. (2020)/Quantitative (2019)	• Observational, retrospective chart review • Data collected from charts between 2006 and 2018 • The BREAST-Q reconstruction module was completed after BRRM to measure satisfaction with breasts	• *N* = 12 • x̄ age at time of BRRM: 26.9 years (range at time of BRRM: 23–29 years) • Location: United States	• BRCA-positive diagnosis • Age under 30 at time of BRRM • Six month since breast reconstruction completed	BREAST-Q reconstruction module/99 items	• Satisfaction with breasts score: 73 • Psychosocial well-being score: 78.2	The BREAST-Q displayed high breast satisfaction and psychosocial well-being scores (out of 100) following BRRM in young patients undergoing nipple-sparing mastectomy
Wasteson et al. (2011)/Qualitative (2011)	• Content analysis • Data originally collected 1993–1997 • One semi-structured interview was conducted per participant to determine long-term consequences of BRRM approximately 10 years after surgery	• *N* = 13 • x̄ age at time of BRRM: 35 years • Location: Sweden	• BRCA-positive diagnosis or increased familiar breast cancer risk • Previous BRRM and study participation	Semi-structured interview guide	Content analysis/6 categories: risk perception, activities after BRRM, spousal bond, recreational activities, cosmetic results, other	• 66% of participants considered cosmetic results to be positive following BRRM • Women would benefit from psychological support after BRRM

### Body Image Findings in Quantitative Literature

The BREAST-Q Reconstruction Module was used to evaluate body image in two studies in this review (Salibian et al., 2020; Gandhi et al., 2021). This instrument is a validated patient-reported outcome measure designed to evaluate outcomes in women who have undergone breast reconstruction. It generates a numerical score on a scale from 0 (worst) to 100 (best), with a higher score indicating a more positive outcome (Mundy et al., 2017). In the BREAST-Q, body image is measured through the satisfaction with breasts and psychosocial well-being domains (Pusic et al., 2017). Participants in both studies expressed high breast satisfaction and psychosocial well-being scores on the BREAST-Q (see Table 1), including a study in which women (*n* = 12) underwent nipple-sparing BRRM before the age of 30 (Salibian et al., 2020). Gandhi et al. (2021) found that women undergoing BRRM (*n* = 241) had higher BREAST-Q scores than the group with breast cancer (*n* = 56). On the satisfaction with breasts scale the BRRM group scored 62 compared to a score of 56 in the cancer group, and on the psychosocial well-being scale the BRRM group score 73, compared to a score of 60 in the cancer group.

In the third study, body image was measured over time with a 31 item study-specific body image scale. It found that satisfaction with breast appearance at the preoperative baseline level was 82.9%. A decline in body image occurred 6 months after BRRM, with only 45.7% of participants reporting satisfaction with breast appearance. At the 21 month follow up body image had improved but remained lower than baseline with 71.4% of women satisfied with breast appearance (Gopie et al., 2013).

#### Breast Cancer Worry and Body Image

Women with a BRCA-positive diagnosis have been found to experience breast cancer- related worry and uncertainty surrounding if and when a breast cancer diagnosis might occur (McQuirter et al., 2010; Hoskins and Greene, 2012; Dean, 2016). Increased breast cancer-related worry was found to lower body image in one study in this review. A study by Gopie et al. (2013) examined if body image could be predicted from breast cancer-related distress using the Impact Event Scale. This scale measures subjective distress related to a specific life event (Horowitz et al., 1979). The study found that a high preoperative cancer distress score led to a more negative body image at long-term follow-up in 25% of study participants (*n* = 12), with a higher preoperative cancer distress score (−0.33) associated with decreased body image at 6 months (Cohen’s *d* = −0.63), and a higher preoperative cancer distress score (0.10) associated with decreased body image at 21 months (Cohen’s *d* = −0.83) (Gopie et al., 2013).

### Body Image Findings in Qualitative Literature

#### The Impact of Psychological Evaluation on Body Image

Braude et al. (2017) have noted that psychologists provide advantages to women considering BRRM, including decision-making and adjustment-preparation pre-operatively and support with adjustment post-operatively. In this review, two qualitative studies also noted the importance of psychological evaluation on the psychosocial outcomes of women in the time surrounding BRRM (Wasteson et al., 2011; Glassey et al., 2018b). In a study where participants underwent BRRM before age 35, those who are able to speak with a psychologist before surgery upheld their confidence, were more satisfied with cosmetic outcomes, and did not develop negative self-esteem postoperatively (Glassey et al., 2018b). However, women who did not undergo evaluation experienced psychosocial adjustment and body image issues after surgery (Glassey et al., 2018b).

#### Unrealistic Expectations

The importance of knowledge and understanding to the change in physical appearance following BRRM appeared to be a key factor in participants forming realistic expectations of breast appearance following surgery (Wasteson et al., 2011; Hallowell et al., 2012; Gopie et al., 2013; Glassey et al., 2018b). One study noted that body image declined postoperatively when women did not receive adequate preparation from their healthcare team on how their reconstructed breasts could appear; to facilitate understanding participants suggested photographs as a helpful medium for visualizing breast reconstruction (Glassey et al., 2018b).

#### Factors Which Improved or Maintained Body Image Following Bilateral Risk-Reducing Mastectomy

Two studies demonstrated that reconstruction following BRRM did not negatively impact body image. In one study, women who were unhappy with their breast appearance preoperatively experienced an improvement in body image due to change in breast shape and size following reconstruction (Glassey et al., 2018b). In a second study, some participants noted a more appealing breast shape, youthful figure, and fit of clothing as positives following reconstruction after BRRM (Hallowell et al., 2012). Nipple preservation appeared to sustain body image as well, with participants who had had nipple-sparing mastectomies reporting their satisfaction through the maintenance of breast appearance (Glassey et al., 2018b).

## Discussion

This literature review aimed to evaluate how body image was impacted following BRRM in young BRCA-positive women. Findings from both quantitative and qualitative studies indicate that body image can be affected both positively and negatively following this risk-reducing surgery. In quantitative studies, body image was measured through both validated and study-specific instruments. A decline in body image was experienced by some women due to breast cancer-related worry, and persisted for many months (Gopie et al., 2013). However, other women were either satisfied with their physical appearance following BRRM or experienced some improvement in body image with the passage of time (Gopie et al., 2013; Salibian et al., 2020; Gandhi et al., 2021).

Qualitative studies in this review found that some participants noted body image decline following BRRM due to insufficient education from their medical team (Glassey et al., 2018b). Information from healthcare providers was necessary to assist women in forming a realistic understanding of physical appearance after surgery. It was also understood that body image declined among some participants when women did not undergo psychological evaluation prior to BRRM (Glassey et al., 2018b). Body image was also found to be sustained or improved following BRRM. This was noted to occur when women were able to preserve their nipples (Glassey et al., 2018b) and in women who were unhappy with their breast appearance preoperatively. These participants expressed improvement in body image BRRM due to reconstruction (Hallowell et al., 2012).

As a weakness in this review, it should be noted that only three articles included samples comprised solely of women less than 40 years of age at the time of BRRM (Glassey et al., 2018b; Salibian et al., 2020). All other studies also included women older than 40 years of age, making it difficult to generalize findings to younger women. Additionally, the body image outcomes in young women who elected not to undergo reconstruction following BRRM were not well-represented in this review. As a limitation of this review, the author had the sole responsibility for the literature search strategy, review of retrieved studies, data elicitation from included studies, and summary of findings. Finally, as the majority of study participants in this review were Caucasian, the homogeneity of the samples should be viewed as a limitation.

Further investigation is needed following BRRM in BRCA-positive young women to further elucidate factors effecting body image. Such efforts are needed to ultimately develop interventions that would improve a negative body image following BRRM in this population. A strength of this review is the discovery of a gap in the literature, with few studies offering the perspective of body image following this risk-reducing surgery in BRCA-positive women less than 40 years of age.

## Conclusion

While a dearth of information exists that focuses exclusively on how young BRCA-positive women perceive their physical appearance after undergoing BRRM, this literature review has identified that younger women experience both positive and negative impacts to body image following this risk-reducing surgery. This echoes findings from studies of body image after BRRM comprised of women across the lifespan (Metcalfe et al., 2015; Razdan et al., 2016; Bai et al., 2019). For clinicians, it is important to appreciate both the positive and negative consequences of BRRM identified in this review when treating young women who are considering, or who have undergone, this risk-reducing surgery to lower lifetime breast cancer risk.

## Author Contributions

The author confirms being the sole contributor of this work and has approved it for publication.

## Conflict of Interest

The author declares that the research was conducted in the absence of any commercial or financial relationships that could be construed as a potential conflict of interest.

## Publisher’s Note

All claims expressed in this article are solely those of the authors and do not necessarily represent those of their affiliated organizations, or those of the publisher, the editors and the reviewers. Any product that may be evaluated in this article, or claim that may be made by its manufacturer, is not guaranteed or endorsed by the publisher.
